# Regional Trends and Variation in Knee Osteotomy Surgery in England, UK: A Longitudinal Analysis of National Health Service Data over Twenty Years

**DOI:** 10.1177/19476035261498345

**Published:** 2026-11-04

**Authors:** Conor Hennessy, Nick Howells, Michael McNicholas, Leela Biant, Simon G.F. Abram

**Affiliations:** 1 Musculoskeletal Research Unit, University of Bristol, Bristol, United Kingdom; 2 Nuffield Department of Orthopaedics, Rheumatology and Musculoskeletal Sciences, University of Oxford, Oxford, United Kingdom; 3 Department of Trauma and Orthopaedics, North Bristol NHS Trust, Bristol, United Kingdom; 4 Department of Trauma and Orthopaedics, Liverpool University Hospitals Foundation Trust, Liverpool, United Kingdom; 5 Department of Trauma & Orthopaedic Surgery, University of Manchester, Manchester, United Kingdom

**Keywords:** knee, osteotomy, trends, variation, epidemiology

## Abstract

**Aims:**

Knee osteotomy is an established joint preserving procedure; however, despite expanding indications and training, national utilisation patterns remain poorly characterized. We investigated long-term temporal trends and geographic variation in the provision of high tibial (HTO) and distal femoral osteotomy (DFO) across the National Health Service (NHS) in England.

**Methods:**

We conducted a retrospective national epidemiological study using NHS Hospital Episode Statistics (HES). All episodes for patients aged 16–80 undergoing knee osteotomy (excluding patellofemoral) between Jan 1, 2002, and Dec 31, 2022, were extracted. Age- and sex-standardised intervention rates were calculated using Office for National Statistics population data. Trends were analysed nationally and regionally by Integrated Care Board (ICB) to determine geographic variation in service provision.

**Results:**

We identified 24,516 cases (22,900 patients) meeting inclusion criteria. The national standardised intervention rate increased by over 300% during the study period, rising from 1.3/100,000 in 2002 to 3.9/100,000 in 2022 (peaking at 5.1/100,000 in 2019). HTO accounted for 71.16% of procedures, and males comprised 60.9% of the cohort. In 2022, intervention rates were highest in the 16–19 (7.3/100,000) and 40–59 (4.8/100,000) age groups. Substantial regional disparity was detected, with a near ten-fold variation in 2022 intervention rates between ICB regions (range: 1.0 to 9.5 per 100,000).

**Conclusion:**

Knee osteotomy utilisation has risen significantly in England, reflecting the growing role of this joint preserving surgery. However, the extreme regional variation suggests a significant potential inequity in access to joint-preservation surgery. These findings highlight an urgent need for the development of standardised treatment guidance and a review of service provision to ensure patients have access to the procedure when indicated.

## Introduction

Knee osteotomy is performed with the goal of altering the weight-bearing biomechanics of the knee to improve joint pain, function, or stability. Indications include the offloading of partial-thickness osteoarthritis, where joint replacement is associated with poor outcomes, or to protect other joint preserving procedures such as complex cartilage, meniscal surgery, or ligament reconstruction. Due to the preservation of the native joint surfaces, osteotomy has a theoretical benefit of better preserving ligament balance, biomechanics, and proprioception.^1^

The management of symptomatic knee osteoarthritis in young and active patients represents a significant therapeutic dilemma and a growing public health challenge. While total knee arthroplasty (TKR) is the gold standard for end-stage disease in older populations, its longevity and functional outcomes are frequently less satisfactory in younger, higher-demand patients.^2^ With the rising burden of osteoarthritis in the working-age population, strategies that preserve the native joint, maintain high activity levels, and delay the need for arthroplasty are of increasing socioeconomic importance. For full-thickness arthritis, especially in young and active patients, it may offer the best chance for these patients to return to a higher level of activity and significantly delay the need for a subsequent arthroplasty.^3-11^

In degenerative joint disease, osteotomy aims to redistribute forces away from the affected compartment and, in appropriately selected patients, may deliver similar outcomes to partial knee replacement.^12,13^ Medial compartment arthritis accounts for over 70% of arthritis around the knee and therefore is the most common compartment to be offloaded.^14^ Osteotomy planning, however, necessitates a thorough assessment of anatomical alignment and joint line obliquity.^15^ Most commonly this is done with a high-tibial osteotomy (HTO), but depending on the person-specific anatomy, distal femoral osteotomy (DFO) or a combined HTO and DFO procedure may be undertaken.^16^

With expanding indications, better evidence and training, the number of patients being offered knee osteotomy is increasing. However, unlike joint replacement surgery, which is subject to intense scrutiny through national registries, the national utilisation of knee osteotomy in the United Kingdom remains poorly characterised. An understanding of the national trends and variations in the use of osteotomy is important to determine the impact of changes in practice. The presence of large geographical variations may suggest an imbalance in provision for patients.

Understanding the epidemiology of this procedure is essential for workforce planning and addressing health inequalities. The aim of this study was to conduct a comprehensive analysis of NHS data to determine the temporal trends, demographic patterns, and regional variation in the provision of high tibial and distal femoral osteotomy in England over a twenty-year period. The appropriate intervention rate for these procedures is not yet known, but the data outlined in this study will aid an assessment of the utilisation of osteotomy nationally, and how this has changed over time.

## Methods

### Data source

The data source was the national Hospital Episode Statistics (HES) dataset for England, UK. HES includes episodes of care delivered in National Health Service (NHS) hospitals, treatment centre care funded by the NHS, and privately funded or non-resident patients treated within NHS England hospitals.^17^

### Procedures

All patients undergoing knee osteotomy (high tibial osteotomy or distal femoral osteotomy) between 1 January 2002 and 31 December 2022 were identified from the recorded Classification of Surgical Operations and Procedures (OPCS-4) codes and extracted from HES for the purposes of this study.

### Statistical analysis

Stata v18.0 (StataCorp, College Station, Texas, USA) was used to perform all analyses. To adjust for changes in population demographics over time, denominator data from the Office for National Statistics (ONS) was used to calculate age and sex standardised rates with 95% confidence intervals, following the methodology of the Association of Public Health Observatories (APHO).^18^

To investigate for any regional variation in practice, the Integrated Care Board (ICB) responsible for each episode of care was identified. In England, 42 ICBs replaced Clinical Commissioning Groups (CCGs) in 2022 and were therefore constructed from prior regional datasets including CCGs for the purposes of this study.^19^ Resident population data by age, sex and year within each ICB was obtained from the ONS. To investigate differences in the ICB of residence and the ICB of treatment, patients were separately mapped to their ICB of residence, and to the ICB in which they were treated. NHS work which was undertaken in private hospitals was mapped to the ICB in which the private hospital resided geographically. Datawrapper (datawrapper Gmbh, Berlin Friedrichshain), was used to map the age-sex standardised rate of surgery by ICB of treatment (region where the surgery was undertaken) and by the ICB of patient residence (region where the patient lived, irrespective of where surgery was undertaken), using the latest ICB boundaries.^19^

### Ethical approval

In accordance with the Health Research Authority (HRA) recommendations on analysis of routinely collected observational data, formal research ethics committee approval was not required for this study.^20^ Prior to release of the data for analysis, however, the project was approved by the NHS Digital Independent Group Advising on the Release of Data (IGARD) committee (NHS Digital application DARS-NIC-667559).

## Results

### Temporal trends

#### Knee Osteotomy

Between 2002 and 2022, 24,516 cases were identified (22,900 patients) meeting the inclusion criteria. Of these, 17,445 (71.16%) were coded as high tibial osteotomy (HTO), 4,181 (17.05%) as distal femoral osteotomy (DFO), and 1,528 (6.23%) were double level osteotomies. A further 1,362 (5.56%) periarticular knee osteotomies were identified but were not more specifically coded. A greater proportion of patients were male (n=14,944, 60.9%), and the intervention rates rose in all age groups. Nationally the age-sex standardised rate of osteotomies increased 3.3-fold from 1.3/100k (95% CI 1.1 to 1.4; n=488) in 2002 to 5.1/100k at their peak in 2019 (95% CI 4.9 to 5.4; n=2,191). Nationally, the rate fell during the COVID-19 pandemic by almost half, to 2.5/100,000 in 2020 (95% CI 2.4 to 2.7; n=1080), and has shown a slow recovery post-pandemic to a rate of 3.9/100k (95% CI 3.7 to 4.1; n=1,698) in 2022 (Figure 1A).

**Figure 1.**
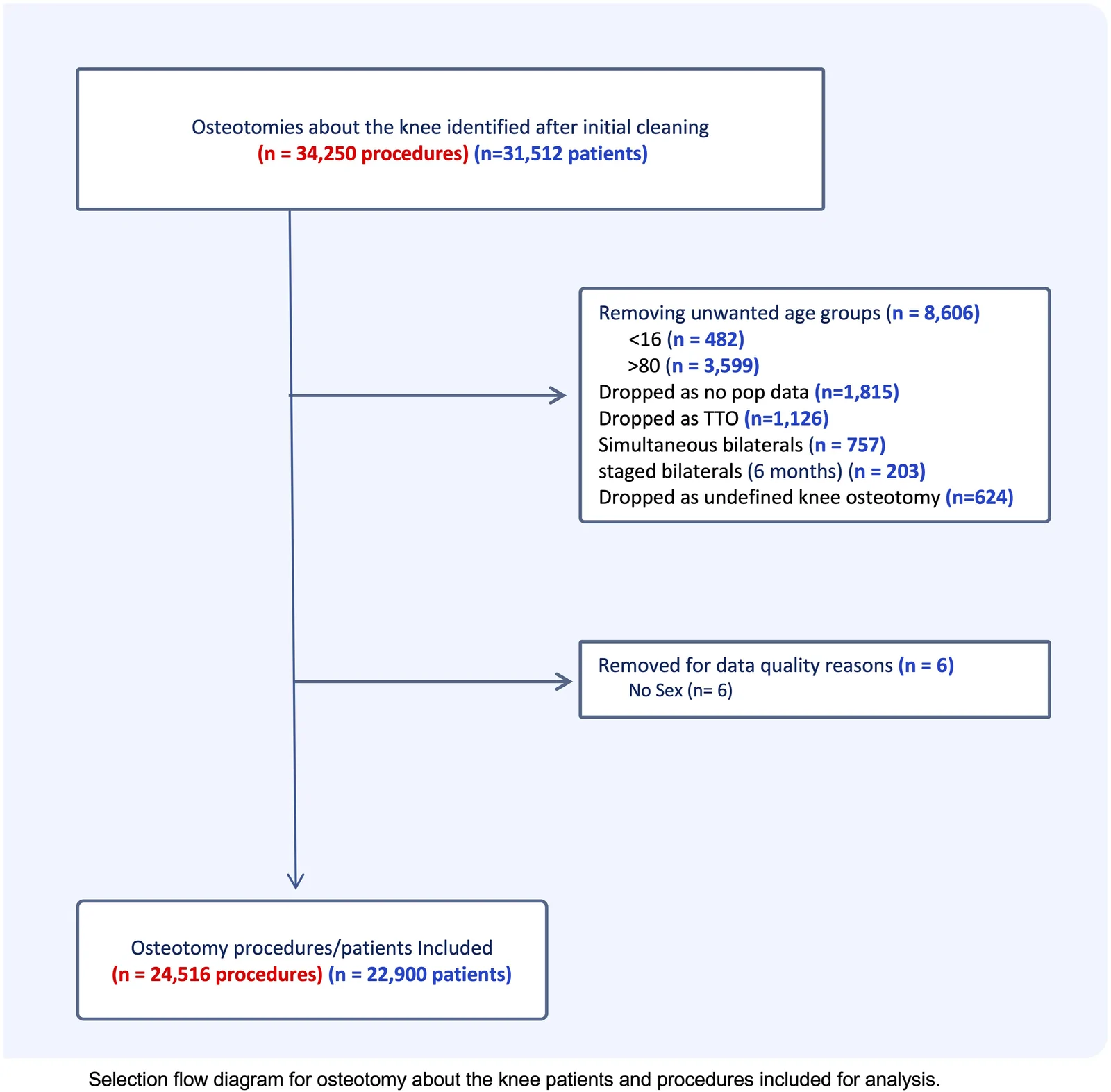
Data selection flow diagram

In 2022, rates were higher in men (4.5 per 100k, (95% CI 4.2 to 4.7 per 100k; n=953) than in women (3.4 per 100k (95% CI 3.2 to 3.6 per 100k; n=745). The highest rates were seen in the 16-19 age group (7.3/100k, (95% CI 6.4 to 8.2) and the 40-59 age group (4.8 per 100k, (95% CI 4.4 to 5.1 per 100k) (Figure 1B).

#### High Tibial Osteotomy

Nationally the age-sex standardised rate of high tibial osteotomies increased 3.85-fold from 0.9/100k (95% CI 0.8 to 1.0; n=348) in 2002 to 3.6/100k at their peak in 2019 (95% CI 3.4 to 3.7; n=1,524). Nationally the rate of HTO fell by almost half to 1.9/100k in 2020 (95% CI 1.8 to 2.0; n=804), and showed a slow recovery post pandemic to a rate of 2.8/100k (95% CI 2.7 to 3.0; n=1,221) in 2022, representing a 3.37-fold increase from the 2002 rate (Figure 1C).

HTO rates were higher in men than women across all years, with the rate in men in 2022 3.2/100k, (95% CI 3.0 to 3.5/100k), 1.5-fold higher than the rate in women (2.4/100k (95% CI 2.2 to 2.6/100k). The highest rates were seen in the 16-19 age group, with a rate of 4.6/100k (95% CI 4.1 to 5.6/100k) seen in this group in 2022 (Figure 1D).

#### Distal Femoral Osteotomy

Nationally the age-sex standardised rate of distal femoral osteotomies increased 4-fold from 0.2/100k (95% CI 0.2 to 0.3; n=93) in 2002 to 1/100k at their peak in 2019 (95% CI 0.9 to 1.1; n=418). Nationally the rate of DFO fell by more than half to 0.4/100k in 2020 (95% CI 0.4 to 0.5; n=180) and showed a slow recovery post pandemic to a rate of 0.7/100k (95% CI 0.6 to 0.7; n=288) in 2022, representing a 3.7-fold increase from the 2002 rate (Figure 1E).

DFO rates were similar in men and women across all years, with the rate in men in 2022 0.7/100k, (95% CI 0.6 to 0.8/100k), and the rate in women 0.7/100k (95% CI 0.5 to 0.8/100k). The highest rates were seen in the 16-19 age group, with a rate of 1.6/100k in 2022 (95% CI 1.2 to 2.1/100k). Similar rates were seen in 20-39 age group (0.7/100k (95% CI 0.6 to 0.9/100k) and 40-59 age group (0.7/100k (95% CI 0.6 to 0.8/100k) in 2022.

#### Double level osteotomies

Double level periarticular osteotomies increased in frequency over the study period. There was no measurable national rate of double level osteotomies in 2002 (0/100k (95% CI 0 to 0; n=8). Only 5 regions showed any measurable activity in 2002, the highest of which was 0.3/100k (95% CI 0.0 - 0.8). The rate of doubles increased to 0.5/100k (95% CI 0.4 to 0.6; n=215) in 2019 and reached a level of 0.4/100k (95% CI 0.3 to 0.4; n=162) in 2022.

### Regional Variation

Regional variation was observed in both the number of osteotomies performed by ICB and the population intervention rate by ICB (Figure 2). In 2022, the highest rate of osteotomy observed was 9.6 knee osteotomies per 100k (95% CI 6.5 to 12.8/100k) (Figure 3B). This represents a rate which is 2.4-fold higher than the national ICB average rate (3.98/100k) in 2022. The lowest rate observed, by ICB, was 1 knee osteotomies/100k (95% CI 0.3 to 1.6/100k) in 2022. In 2022 the national average rate was 3.98/100k, and 4 ICBs performed more than twice the national average rate of knee osteotomies. A different pattern was observed in the absolute number of osteotomies performed in each region. The highest number of knee osteotomy procedures done in an ICB, in 2022, was 180, and the lowest number was 9. There were also differences in the intervention rate according to where patients lived in comparison to where they were treated, confirming that many patients travel between ICB regions or from regions outside the NHS in England, to have a knee osteotomy (Figure 5). The difference between the ICB of residence and ICB of treatment for knee osteotomies was examined, with the difference across the regions being calculated and represented as the procedure delta. In 2022 the highest delta was +70, with the lowest being –46.

**Figure 2.**
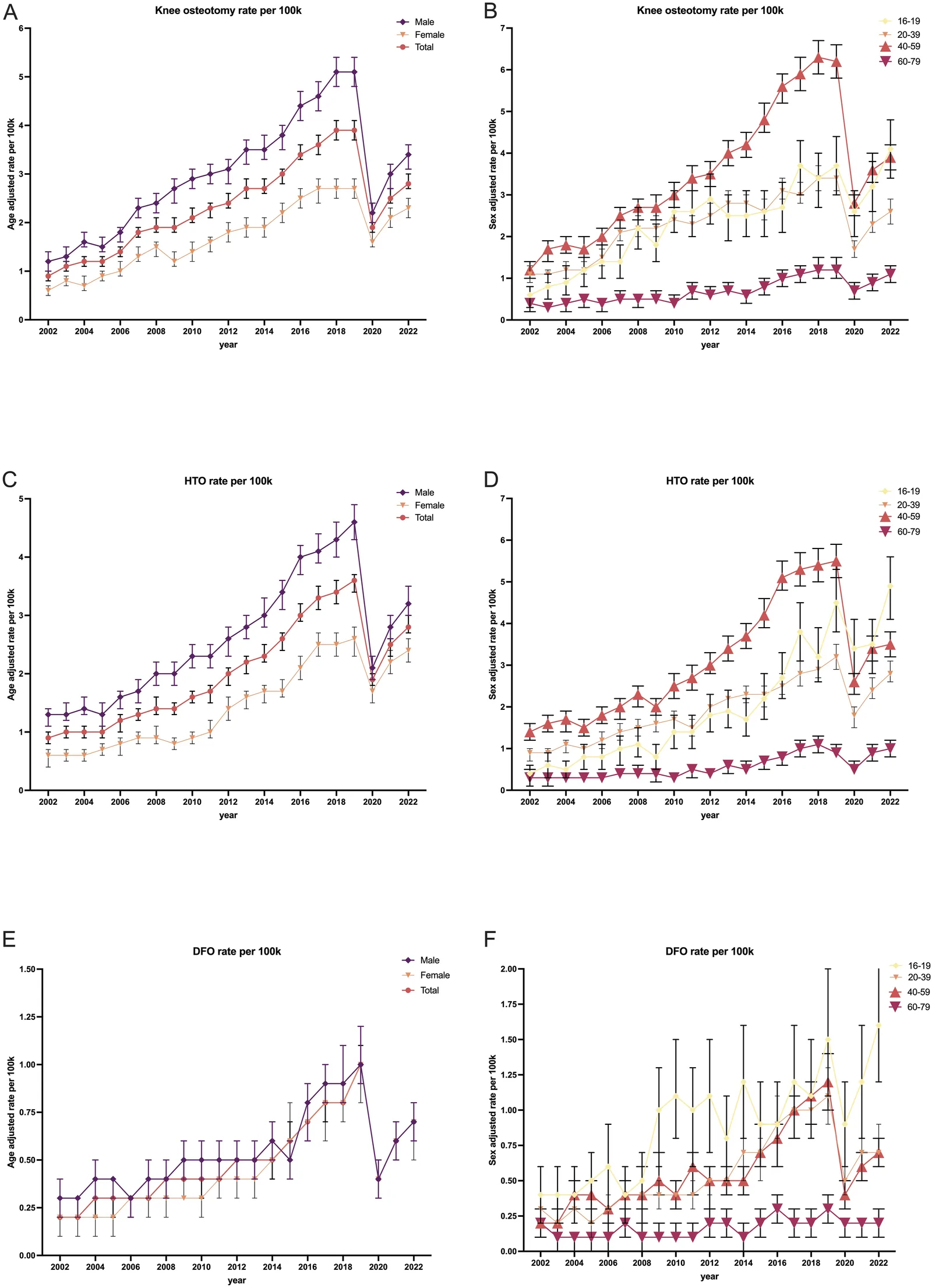
Trends in population rates of osteotomy by age and sex over the study period

**Figure 3.**
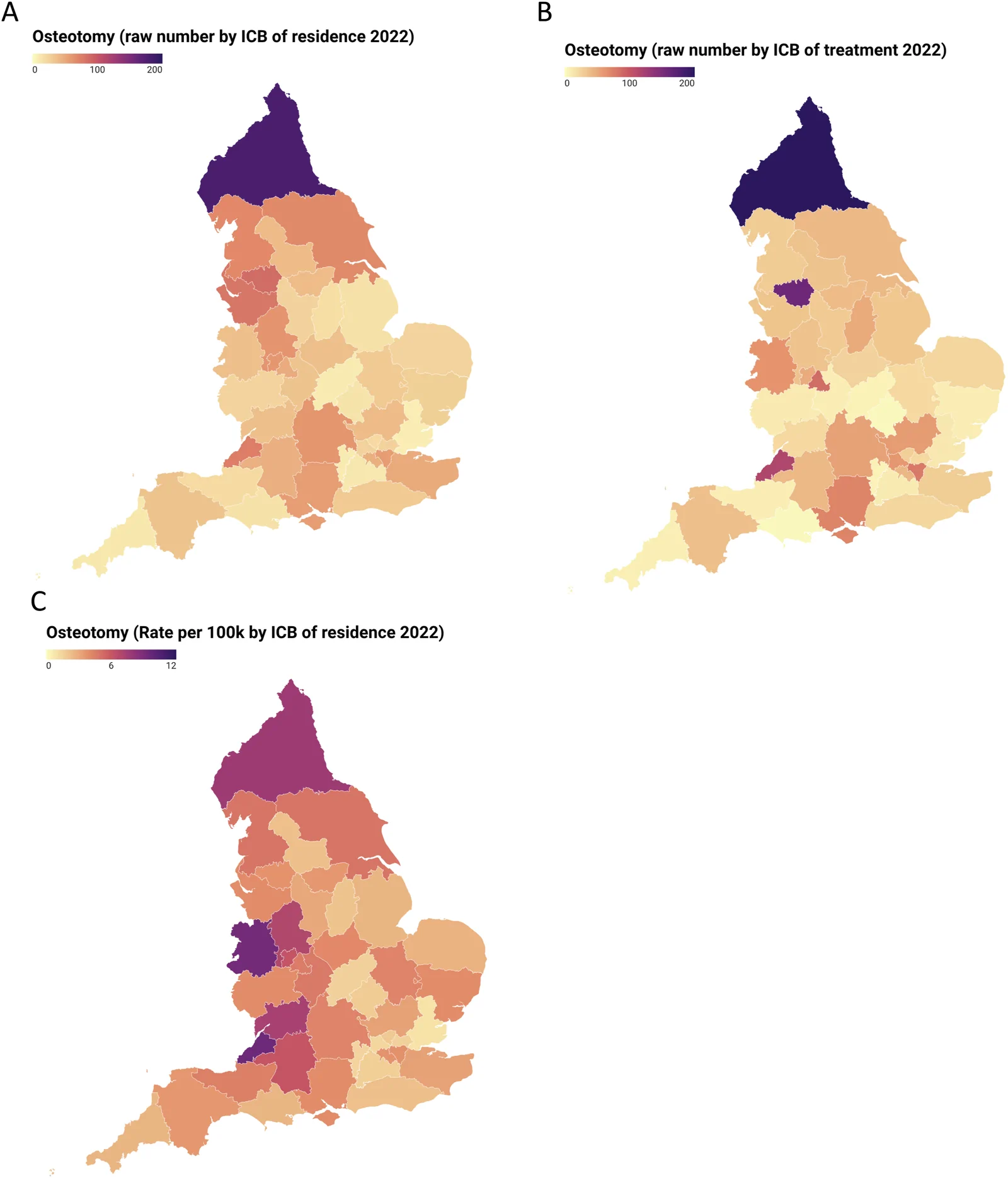
National heat map of knee osteotomy intervention rates Data shown is raw number of procedures (A, B) and age and sex standardised rates per 100,000 (C) of the population. Regions shown are integrated care boards (ICBs)

### Surgeon Volumes

Individual surgeon level volume was obtained from HES data, with surgeons identified by a pseudo-anonymised code. The average number of osteotomies performed among all surgeons who performed at least one osteotomy in 2022, was 4.20. The twenty highest volume knee osteotomy surgeons performed approximately four-times the national average number of procedures in 2022 (>17 osteotomies/year).

## Discussion

### Temporal trends

Between 2002 and 2022, the age-sex standardised rate of knee osteotomy increased 3.54-fold. However, the rate in 2022 (3.9/100,000) remained lower than the 2019 peak (5.1/100,000). The underlying reasons for the overall increase and the more recent post-pandemic decline are uncertain. Potential factors include appropriate adjustments in intervention rates from previous under-provision, increasing presentations, surgeon numbers, specialist training, changes in healthcare commissioning, or evolving patient treatment preferences. The option to defer arthroplasty, especially in younger more active patients may drive more surgeons towards joint preservation with the goal of maintaining activity and preserving function. A California population based study showed a 4.7 fold higher risk of early failure of arthroplasty in patients under 50 compared to those over 65,^21^ and a Scottish arthroplasty registry study showed significantly higher lifetime revision risk in patients aged 16-49.^22^ These findings coupled with poor return to high impact activity and lower satisfaction in younger arthroplasty cohorts may explain the increased utilisation of osteotomy.^23,24^ Other potential reasons for the increases seen could be expanding interest in non-OA indications for osteotomy. The use of osteotomy for instability as an isolated procedure or in combination with ligament reconstruction has seen expanded interest, particularly in the correction of coronal malalignment and posterior tibial slope correction in revision ACL reconstruction.^24,25^ Furthermore, in younger, highly-active individuals, such as athletes or military personnel, osteotomy may be used effectively to regain and preserve demanding functional requirements.^26^

The trends observed in England contrast with the patterns reported in several international studies. In Sweden, the osteotomy rate declined from 5/100,000 in 2008 to 2.3/100,000 in 2023, averaging approximately 296 procedures per year.^27^ The most common age groups were 45-54 years, followed by 15-24 years, with a high degree of regional variation.^27^ Similarly, in Italy, the rate decreased from 4.5/100,000 in 2002 to 2.7/100,000 in 2016, representing approximately 1500-2000 procedures per year.^28^ In the United States, an analysis of the PearlDiver database also indicated a declining trend in osteotomy numbers and evidence was presented that suggested this might be associated with increased utilisation of unicompartmental knee replacement.^29^ For instance, in 2020, only 105 HTO cases were identified in the database compared to 140,593 total knee arthroplasties and 8,652 unicompartmental replacements. In France, annual volumes fell from 6,426 in 2011 to 2,005 in 2020, representing an intervention rate of approximately 3/100,000.^30^ Finally, in Finland, rates fell significantly between 1986 and 2008 to approximately 3/100,000.^31^ The authors suggested that improved arthroplasty outcomes narrowed osteotomy indications; however, they notably observed an increasing trend in patients under 50 years old.^31^ These findings may reflect the differences in UKA usage in different health systems, although direct comparisons remain difficult due to the lack of dedicated osteotomy registries. US data suggests UKA far outweighs osteotomy usage, with data from 2007 – 2021 suggesting a 14 fold difference in UKA versus osteotomy utilisation.^32,33^ Data from the UK suggests UKA usage grew from ∼8% in 2009 to 14% over the following decades, and is maintained at this level, with over 85% of arthroplasty continuing to be TKA^34,35^; our data suggests a rising role for osteotomy but with consideration regional variation.

### Regional variation

Comparable regional variation has been reported for shoulder, hip, and knee arthroscopy in England.^36-39^ Factors underlying such variation include service or population differences, clinician beliefs, surgeon volume, and technical improvements broadening accessibility to non-specialists.^40^ Strategies to address unjustified variation include education, treatment guidelines, and providing clinician feedback to facilitate peer comparison.^41^ Furthermore, ‘appropriateness criteria’ have been used successfully in other procedures to standardise decisions and identify overuse.^42^

We recorded higher rates of knee osteotomy in specialist tertiary referral centres. By examining the difference between the ICB of treatment and residence, we noted significant patient migration in many regions. These patterns appear to represent movement towards centralised specialist centres, with the ICB of residence often being those surrounding the treatment hub. This finding highlights the importance of referral networks in supporting regional provision and may inform future investigations into adequate unit-level and surgeon-level volumes.

#### Age-related variation

Our study observed high rates of osteotomy in the 16-19 cohort (7.3/100k in 2022). This peak may represent the optimum timing for corrective osteotomy in young adults with residual deformities as a result of congenital, developmental or metabolic malalignment. The intervention rate observed in this age group may include a significant proportion of procedures deferred to allow complete physeal closure. A German nationwide database study showed similar results, with the highest osteotomy rates seen in the under 25 age group.^43^ A study from 2010 suggested that HTO in patients under 25 produces good function outcomes and may prevent future osteoarthritic change.^44^ Thus, our observation of high rates in this young age group may reflect surgeon specific selection of patients who have reached adequate skeletal maturity for osteotomy, and the evidence of benefit from early correction to prevent future osteoarthritic change.

#### Gender differences

In our study cohort we observed that HTO was more commonly performed in male patients, with a 1.5 fold higher rate seen compared to female patients, with DFO rates being similar across genders. This finding was in keeping with a German registry study which showed over twice the rate of HTO in men compared to women (RR 2.43), with DFO being less common and having a female predominance.^43^ US data showed similar findings, with HTO being more frequent in younger male patients, and DFO once again more common in older female patients.^45^ The male predominance in knee osteotomy may be driven by the fact that HTO for medical compartment varus misaligned knees represents the highest volume osteotomy, and varus malalignment with early medial compartment overload is predominantly found in young active men.^46^

#### Inequity of access to osteotomy

Contemporary UK national joint registry (NJR) data indicate that over 3,600 surgeons are performing knee arthroplasty annually, with in excess of 100,000 procedures performed each year.^47^This compares to approximately 1700 annual procedures done by 420 surgeons observed in this study, an annual volume of osteotomy <1% of the volume of joint replacements, with practice concentrated in specialist centres with higher-volume surgeons. These findings are consistent with the marked regional variation and patient migration demonstrated in our study (Figures 3-5), suggesting access to osteotomy is not uniform across England. The specialised nature of osteotomy may mean patients require referral to tertiary centres, potentially in regions significant distances from their area of residence. While centralisation may be appropriate for technically demanding procedures such as osteotomy, the substantial geographic variation raises the possibility that access may depend in part on where patients live and the availability of referral pathways.

**Figure 4.**
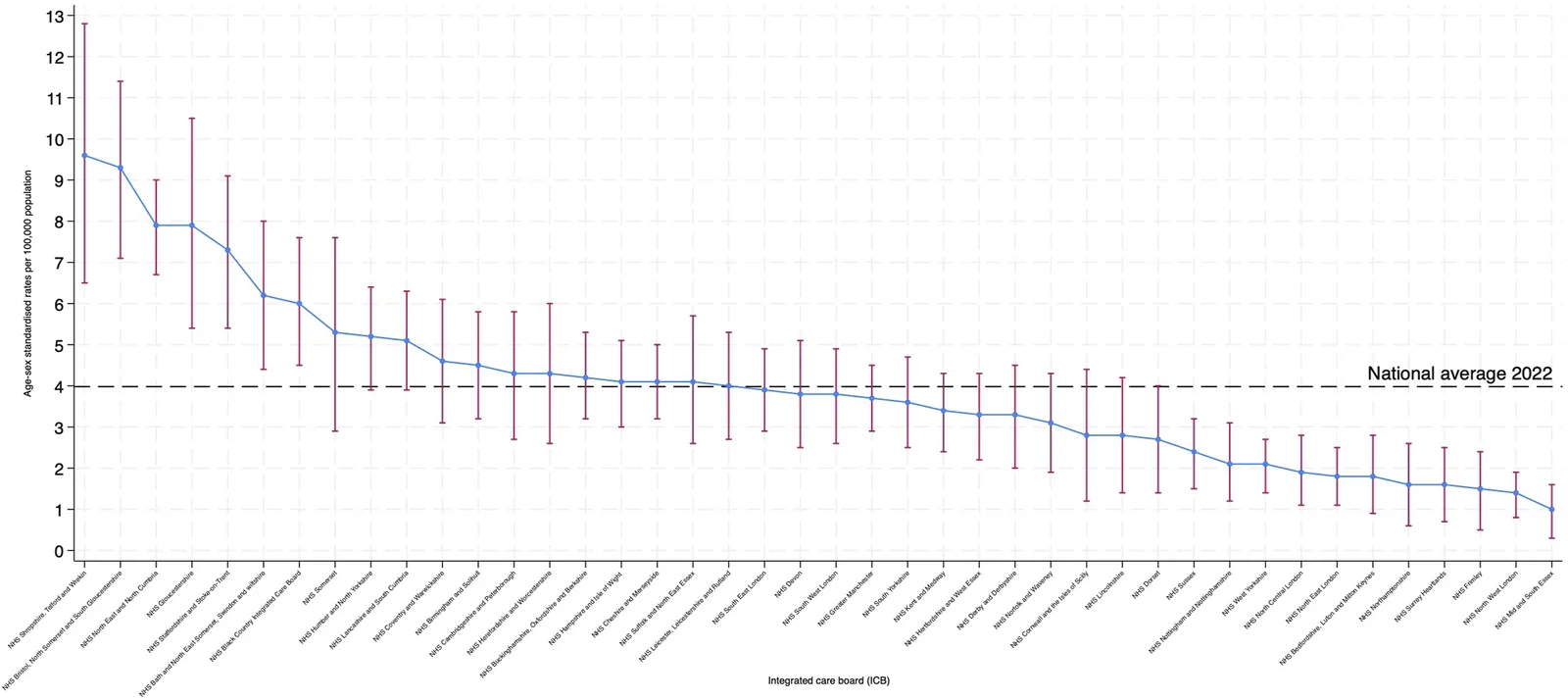
Osteotomy rates per 100,000 across the 42 NHS England Integrated Care Boards (ICB of patient residence) in 2022

**Figure 5.**
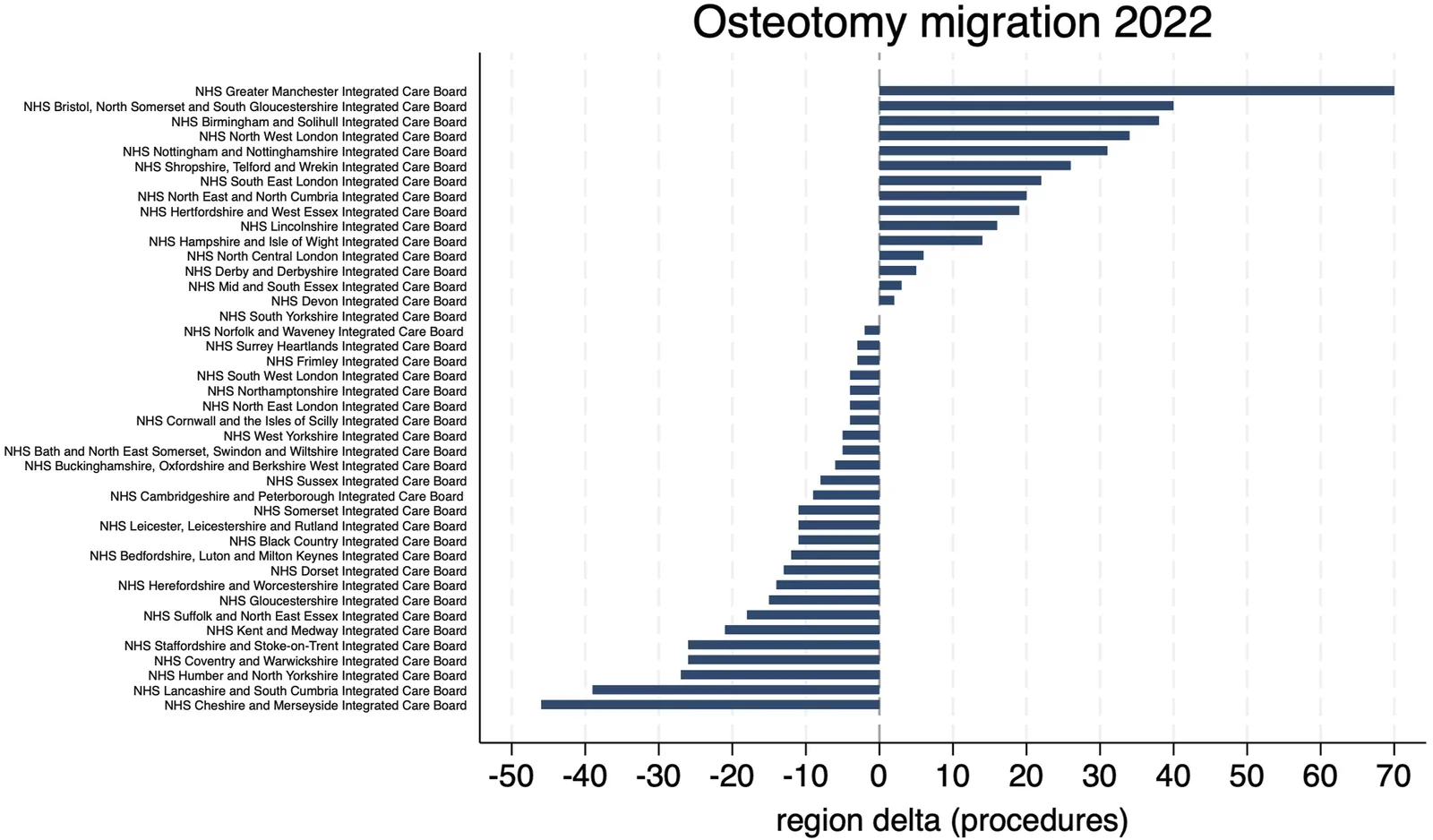
Migration between Integrated Care Boards Data shown is the difference between the raw number of osteotomies by ICB of residence in a given region, and raw number by ICB of treatment. A positive (right) skew representing patients migrating into a region for the procedure and a negative (left skew) representing patients migrating out of the region for the procedure

Technological developments may help reduce some of the technical barriers to wider adoption. Patient specific instrumentation, computer assisted navigation and newer generation locking plates have been associated with improved accuracy of alignment correction and more stable fixation.^48-50^ Advances in pre-operative planning and techniques that reduce variability may therefor enable a broader group of appropriately trained surgeons to incorporate osteotomy within contemporary joint preservation practice, Nevertheless, ensuring equitable access is likely to require not only technological access, but also appropriate training. The low numbers of consultants performing the procedure nationally likely results in significant differences in exposure to osteotomy among trainee orthopaedic surgeons. Clinicians with limited exposure in training or fellowship to osteotomy may be less familiar with its indications and as such less comfortable referring patients for the procedure, especially if it is not openly available locally. Consequently, suitable patients may never reach specialist assessment or be offered osteotomy even if it would be appropriate or favourable. Greater awareness of indications, clearer regional or interregional referral pathways and consideration of the organisation of osteotomy services nationally may therefore be appropriate to reduce unwanted variation in access.

### Strengths and limitations

There are some limitations to our study. These data are observational only and the causative factors underlying the observed intervention rates cannot be determined. Hospital Trusts rely on the accuracy of HES data reporting for payment for the surgical procedures performed and this provides a strong incentive for procedural coding data accuracy, but the absolute accuracy cannot be proven. Although the specific codes used in this study have not been formally validated, for reference, HES comorbidity field coding and national arthroplasty registry data have both been shown to correlate strongly with primary care records.^51,52^

Due to the distribution of hospitals and service, some ICBs commission services including osteotomy outside of the usual boundaries. Regional treatment rates may, therefore, be over- or under-estimated when there is a high level of migration in or out of one region to another. Both the ICB of patient treatment and the ICB of patient home residence is reported in this study alongside migration figures for completeness and to ease interpretation of the potential impact of these factors.

It is important to note that HES does not include private sector procedures, unless funded by the NHS or privately funded but performed in an NHS hospital. The number of knee osteotomy procedures performed in the private healthcare sector is unknown but private healthcare expenditure as a proportion of total private and public expenditure has remained relatively stable over time, suggesting this is unlikely to explain the observed changes in the rate of surgery in England.

## Conclusion

Knee osteotomy rates in England increased significantly over the last 20 years, though intervention rates have plateaued or fallen slightly since the COVID-19 pandemic. Despite this overall increase, a high degree of regional variation persists, suggesting potential inequity of access. Although the determinants of this variation cannot be established from administrative data, the concentration of osteotomy practice in specialist surgical centres suggests that local expertise, training and established referral pathways may be important factors. Improving the awareness of the indications for osteotomy, developing clearer regional and inter-regional referral pathways and considering the national organisation of specialist osteotomy services may reduce variation and support more equitable access to osteotomy. Finally, development of mandatory registries for osteotomy would facilitate more robust monitoring of indications, outcomes, and practice patterns, helping to define better patient selection criteria and inform future service provision.

## Data Availability

The data used in this study is the property of NHS digital. The data that support the findings of this study are available from NHS Digital but restrictions apply to the availability and sharing of this data, which were used under license for the current study, and so are not publicly available. *

## References

[1] Bowd J Van Rossom S Williams D , et al. Using musculoskeletal modelling to estimate knee joint loading pre and post high tibial osteotomy. Clin Biomech Bristol Avon. 2023;101:105855. 10.1016/j.clinbiomech.2022.105855 36563543

[2] Bayliss LE Culliford D Monk AP , et al. The effect of patient age at intervention on risk of implant revision after total replacement of the hip or knee: a population-based cohort study. Lancet Lond Engl. 2017;389:1424-1430. 10.1016/S0140-6736(17)30059-4 PMC5522532 28209371

[3] Berman AT Bosacco SJ Kirshner S Avolio A . Factors influencing long-term results in high tibial osteotomy. Clin Orthop. 1991:192-198. 1934732

[4] Brouwer RW Huizinga MR Duivenvoorden T , et al. Osteotomy for treating knee osteoarthritis. Cochrane Database Syst Rev. 2014;2014:CD004019. 10.1002/14651858.CD004019.pub4 PMC7173694 25503775

[5] Cameron HU Botsford DJ Park Y-S . Prognostic factors in the outcome of supracondylar femoral osteotomy for lateral compartment osteoarthritis of the knee. Can J Surg. 1997;40:114-119. PMC3952972 9126124

[6] Finkelstein JA Gross AE Davis A . Varus osteotomy of the distal part of the femur. A survivorship analysis. J Bone Joint Surg Am. 1996;78:1348-1352. 10.2106/00004623-199609000-00008 8816649

[7] Hernigou P Medevielle D Debeyre J Goutallier D . Proximal tibial osteotomy for osteoarthritis with varus deformity. A ten to thirteen-year follow-up study. J Bone Joint Surg Am. 1987;69:332-354. 3818700

[8] Mathews J Cobb AG Richardson S Bentley G . Distal femoral osteotomy for lateral compartment osteoarthritis of the knee. Orthopedics. 1998;21:437-440. 10.3928/0147-7447-19980401-08 9571677

[9] Naudie D Bourne RB Rorabeck CH Bourne TJ . The Install Award. Survivorship of the high tibial valgus osteotomy. A 10- to -22-year followup study. Clin Orthop. 1999;367:18-27. 10546594

[10] van Raaij TM Reijman M Furlan AD Verhaar JAN . Total knee arthroplasty after high tibial osteotomy. A systematic review. BMC Musculoskelet Disord. 2009;10:88. 10.1186/1471-2474-10-88 PMC2720911 19619275

[11] Rudan JF Simurda MA . High tibial osteotomy. A prospective clinical and roentgenographic review. Clin Orthop. 1990;255:251-256. 2347159

[12] Ollivier M Kley K Pareek A Parratte S Hirschmann MT . Critical considerations in the selection between knee osteotomy and unicompartmental knee arthroplasty in younger patients with varus alignment and early-stage knee osteoarthritis. Knee Surg Sports Traumatol Arthrosc Off J ESSKA. 2025;33:3445-3453. 10.1002/ksa.12801 40679265

[13] Jacquet C Gulagaci F Schmidt A , et al. Opening wedge high tibial osteotomy allows better outcomes than unicompartmental knee arthroplasty in patients expecting to return to impact sports. Knee Surg Sports Traumatol Arthrosc Off J ESSKA. 2020;28:3849-3857. 10.1007/s00167-020-05857-1 32008058

[14] Slemenda CW . The epidemiology of osteoarthritis of the knee. Curr Opin Rheumatol. 1992;4:546-551. 1503880

[15] Dawson M Elson D Claes S , et al. Osteotomy around the painful degenerative varus knee has broader indications than conventionally described but must follow a strict planning process: ESSKA Formal Consensus Part I. Knee Surg Sports Traumatol Arthrosc Off J ESSKA. 2024;32:1891-1901. 10.1002/ksa.12256 38738832

[16] Ollivier M Claes S Mabrouk A , et al. Surgical strategy and complication management of osteotomy around the painful degenerative varus knee: ESSKA Formal Consensus Part II. Knee Surg Sports Traumatol Arthrosc Off J ESSKA. 2024;32:2194-2205. 10.1002/ksa.12273 38769785

[17] Hospital Episode Statistics (HES). https://digital.nhs.uk/data-and-information/data-tools-and-services/data-services/hospital-episode-statistics

[18] APHO . Commonly used public health statistics and their confidence intervals. Public Health England; 2010. https://fingertips.phe.org.uk/profile/guidance.

[19] NHS England . Integrated care boards in England. https://www.england.nhs.uk/publication/integrated-care-boards-in-england/. accessed Oct 25, 2024.

[20] HRA Decision Tool. https://www.hra-decisiontools.org.uk/research/ (accessed Oct 25, 2024).

[21] Meehan JP Danielsen B Kim SH Jamali AA White RH . Younger age is associated with a higher risk of early periprosthetic joint infection and aseptic mechanical failure after total knee arthroplasty. J Bone Joint Surg Am. 2014;96:529-535. 10.2106/JBJS.M.00545 24695918

[22] Morrison SR Hall AJ Lim B Akhtar A Walmsley P . Knee arthroplasty in patients under 50 years demonstrates durable survivorship despite substantially higher lifetime revision risk than in older patients. Knee Surg Sports Traumatol Arthrosc Off J ESSKA. 2026;epub ahead of print:70486. doi:10.1002/ksa.70486. published online June 15. 42290641

[23] Bergstein VE Weinblatt AI Taylor WL Long WJ . Total knee arthroplasty survivorship and outcomes in young patients: a review of the literature and 40-year update to a longitudinal study. Arch Orthop Trauma Surg. 2024;144:4077-4083. 10.1007/s00402-024-05198-5 PMC11564207 38436715

[24] Hoorntje A Witjes S Kuijer PPFM , et al. High Rates of Return to Sports Activities and Work After Osteotomies Around the Knee: A Systematic Review and Meta-Analysis. Sports Med. 2017;47:2219-2244. 10.1007/s40279-017-0726-y PMC5633634 28401426

[25] Klek M Dhawan A . The Role of High Tibial Osteotomy in ACL Reconstruction in Knees with Coronal and Sagittal Plane Deformity. Curr Rev Musculoskelet Med. 2019;12:466-471. 10.1007/s12178-019-09589-9 PMC6942054 31760623

[26] Klasér J Kotake L Lindberg M , et al. Prevalence of osteoarthritis and clinical outcomes in patients with fractures of the tibial plateau - medium- and long-term analysis. BMC Musculoskelet Disord. 2025;26:522. 10.1186/s12891-025-08786-7 PMC12107934 40426131

[27] Nilsson G Schmidt V Axenhus M . Decreasing utilization of tibial osteotomy and regional disparities: A population-based study of all surgeries in Sweden from 2008 to 2023. J Exp Orthop. 2025;12:e70386. 10.1002/jeo2.70386 PMC12296692 40718549

[28] Longo UG Mazzola A Campi S , et al. Annual Trends of High Tibial Osteotomy: Analysis of an Official Registry in Italy. Medicina (Mex). 2024;60:1168. 10.3390/medicina60071168 PMC11279272 39064599

[29] Collins LK Waters TL Cole MW , et al. Incidence and Trends of High Tibial Osteotomy and Unicompartmental Knee Arthroplasty Over the Past Decade: A Lost Art. Arthroplasty Today. 2023;20:101121. 10.1016/j.artd.2023.101121 PMC10014255 36938354

[30] Fitoussi A Dartus J Erivan R , et al. Management of medial femorotibial osteoarthritis: Epidemiology, and survival of unicompartmental knee arthroplasty versus valgus high tibial osteotomy in France. Study of 108,007 cases from the French National Hospitals Database. Orthop Traumatol Surg Res. 2023;109:103692. 10.1016/j.otsr.2023.103692 37776952

[31] Niinimäki TT Eskelinen A Ohtonen P Junnila M Leppilahti J . Incidence of osteotomies around the knee for the treatment of knee osteoarthritis: A 22-year population-based study. Int Orthop. 2012;36:1399-1402. 10.1007/s00264-012-1508-7 PMC3385883 22354471

[32] Kazarian GS Lonner JH Maltenfort MG Ghomrawi HMK Chen AF . Cost-Effectiveness of Surgical and Nonsurgical Treatments for Unicompartmental Knee Arthritis: A Markov Model. J Bone Joint Surg Am. 2018;100:1653-1660. 10.2106/JBJS.17.00837 30277995

[33] Debopadhaya S Acosta E Ortiz D . Trends and outcomes in the surgical management of young adults with knee osteoarthritis using high tibial osteotomy and unicompartmental knee arthroplasty. Arch Orthop Trauma Surg. 2024;144:3995-4002. 10.1007/s00402-024-05362-x 38771360

[34] Ma X Wang Y Fu K . High tibial osteotomy versus unicompartmental knee arthroplasty for medial compartment knee osteoarthritis: a comprehensive meta-analysis with patient-specific stratification by age, BMI, and preoperative activity levels. BMC Musculoskelet Disord. 2026;27:270. 10.1186/s12891-026-09590-7 PMC13041129 41735997

[35] Beard DJ Davies LJ Cook JA , et al. The clinical and cost-effectiveness of total versus partial knee replacement in patients with medial compartment osteoarthritis (TOPKAT): 5-year outcomes of a randomised controlled trial. Lancet. 2019;394:746-756. 10.1016/S0140-6736(19)31281-4 PMC6727069 31326135

[36] Valsamis EM Pinedo-Villanueva R Sayers A Collins GS Rees JL . Shoulder replacement surgery’s rising demand, inequality of provision, and variation in outcomes: cohort study using Hospital Episode Statistics for England. BMC Med. 2023;21:406. 10.1186/s12916-023-03112-1 PMC10601312 37880689

[37] Matharu G Culliford D Blom A Judge A . Projections for primary hip and knee replacement surgery up to the year 2060: an analysis based on data from The National Joint Registry for England, Wales, Northern Ireland and the Isle of Man. Ann R Coll Surg Engl. 2022;104:443-448. 10.1308/rcsann.2021.0206 PMC9157920 34939832

[38] Abram SGF Judge A Beard DJ Wilson HA Price AJ . Temporal trends and regional variation in the rate of arthroscopic knee surgery in England: analysis of over 1.7 million procedures between 1997 and 2017. Has practice changed in response to new evidence? Br J Sports Med. 2018;53:1533-1538. bjsports-2018-099414. 10.1136/bjsports-2018-099414 30279217

[39] Abram SGF Price AJ Judge A Beard DJ . Anterior cruciate ligament (ACL) reconstruction and meniscal repair rates have both increased in the past 20 years in England: hospital statistics from 1997 to 2017. Br J Sports Med. 2020;54:286-291. 10.1136/bjsports-2018-100195 30661013

[40] Birkmeyer JD Reames BN McCulloch P Carr AJ Campbell WB Wennberg JE . Understanding of regional variation in the use of surgery. The Lancet. 2013;382:1121-1129. 10.1016/S0140-6736(13)61215-5 PMC4211114 24075052

[41] Mcculloch P Nagendran M Campbell WB , et al. Variation in Surgery 2 Strategies to reduce variation in the use of surgery. The Lancet. 2013;382:1130-1139. 10.1016/S0140-6736(13)61216-7 24075053

[42] Lawson EH Gibbons MM Ko CY Shekelle PG . The appropriateness method has acceptable reliability and validity for assessing overuse and underuse of surgical procedures. J Clin Epidemiol. 2012;65:1133-1143. 10.1016/j.jclinepi.2012.07.002 23017632

[43] Domnick C Garcia P Raschke MJ , et al. Trends and incidences of ligament-surgeries and osteotomies of the knee: an analysis of German inpatient records 2005–2013. Arch Orthop Trauma Surg. 2017;137:989-995. 10.1007/s00402-017-2704-0 28466182

[44] Masrouha KZ Sraj S Lakkis S Saghieh S . High tibial osteotomy in young adults with constitutional tibia vara. Knee Surg Sports Traumatol Arthrosc Off J ESSKA. 2011;19:89-93. 10.1007/s00167-010-1148-7 20411373

[45] Delva ML Samuel LT Roth A Yalçin S Kamath AF . Contemporary Knee Osteotomy in the United States: High Tibial Osteotomy and Distal Femoral Osteotomy Have Comparable Complication Rates despite Differing Demographic Profiles. J Knee Surg. 2021;34:816-821. 10.1055/s-0039-3400742 31777032

[46] Hunter DJ Bierma-Zeinstra S . Osteoarthritis. Lancet. 2019;393:1745-1759. 10.1016/S0140-6736(19)30417-9 31034380

[47] National Joint Registry . (2025). The National Joint Registry 22nd annual report 2025: Executive summary. National Joint Registry. Retrieved August 24, 2026, from. https://reports.njrcentre.org.uk/. https://reports.njrcentre.org.uk/AR-Executive-Summary.

[48] Aman ZS DePhillipo NN Peebles LA Familiari F LaPrade RF Dekker TJ . Improved Accuracy of Coronal Alignment Can Be Attained Using 3D-Printed Patient-Specific Instrumentation for Knee Osteotomies: A Systematic Review of Level III and IV Studies, 38. Arthrosc J Arthrosc Relat Surg Off Publ Arthrosc Assoc N Am Int Arthrosc Assoc; 2022:2741-2758. 10.1016/j.arthro.2022.02.023 35247513

[49] Reising K Strohm PC Hauschild O , et al. Computer-assisted navigation for the intraoperative assessment of lower limb alignment in high tibial osteotomy can avoid outliers compared with the conventional technique. Knee Surg Sports Traumatol Arthrosc Off J ESSKA. 2013;21:181-188. 10.1007/s00167-012-2088-1 22760216

[50] Zaffagnini S Dal Fabbro G Belvedere C , et al. Custom-Made Devices Represent a Promising Tool to Increase Correction Accuracy of High Tibial Osteotomy: A Systematic Review of the Literature and Presentation of Pilot Cases with a New 3D-Printed System. J Clin Med. 2022;11:5717. 10.3390/jcm11195717 PMC9571741 36233583

[51] Crooks CJ West J Card TR . A comparison of the recording of comorbidity in primary and secondary care by using the Charlson Index to predict short-term and long-term survival in a routine linked data cohort. BMJ Open. 2015;5:1-9. 10.1136/bmjopen-2015-007974 PMC4458584 26048212

[52] Boulton C Harrison C Wilton T , et al. Implementing large-scale data quality validation in a national arthroplasty registry to improve compliance: the National Joint Registry data quality audit programme. Bone Jt Open. 2022;3:716-725. 10.1302/2633-1462.39.BJO-2022-0051.R1 PMC9533243 36106695

